# Biological Aging and Chemotoxicity in Patients with Colorectal Cancer: A Secondary Data Analysis Using EHR Data

**DOI:** 10.3390/curroncol32080438

**Published:** 2025-08-05

**Authors:** Claire J. Han, Ashley E. Rosko, Jesse J. Plascak, Alai Tan, Anne M. Noonan, Christin E. Burd

**Affiliations:** 1 Center for Healthy Aging, Self-Management and Complex Care, The Ohio State University College of Nursing, The Ohio State University–James: Cancer Treatment, and Research Center, Columbus, OH 43210, USA; 2 Division of Hematology, The Ohio State University, Columbus, OH 43210, USA; ashley.rosko@osumc.edu; 3 Division of Cancer Prevention and Control, Ohio State University College of Medicine, The Ohio State University–James: Comprehensive Cancer Center, Columbus, OH 43210, USA; jesse.plascak@osumc.edu; 4 Center for Healthy Aging, Self-Management, and Complex Care, Office of Research, Biostatistics, The Ohio State University College of Nursing, Columbus, OH 43210, USA; tan.739@osu.edu; 5 GI Medical Oncology Section, The Ohio State University–James: Cancer Treatment and Research Center, Columbus, OH 43210, USA; anne.noonan@osumc.edu; 6 Departments of Molecular Genetics, Cancer Biology and Genetics, The Ohio State University, Columbus, OH 43210, USA; christin.burd@osumc.edu

**Keywords:** biological aging, age acceleration, risk factors, chemotoxicity, colorectal cancer, Levine Phenotypic Age, electronic health records

## Abstract

**Background**: Biological aging influences cancer outcomes, but its changes during chemotherapy and impact on chemotoxicity in colorectal cancer (CRC) remain underinvestigated. We examined (1) trajectories of biological aging (using Levine Phenotypic Age) during six months of chemotherapy, (2) sociodemographic and clinical risk factors for biological aging, and (3) links between biological aging and chemotoxicity. **Methods:** Using data from electronic health records (2013–2019) from 1129 adult CRC patients, we computed biological aging (raw Levine Phenotypic Age and its age acceleration [Levine Phenotypic Age–chronological age]) from routine blood tests (e.g., complete blood counts, hepatorenal/inflammatory markers). Chemotoxicity was identified primarily via International Classification of Diseases (ICD-9 and -10) codes. **Results:** Chemotherapy accelerated biological aging over time. Biological aging at baseline and changes over time predicted chemotoxicity. However, changes in biological aging over time showed stronger associations than baseline biological aging. Advanced cancer stages, higher comorbidity burden, and socioeconomic disadvantage (especially area-level deprivation) were associated with accelerated biological aging at baseline and over time. Biological aging occurred across both young and older adults. **Conclusions:** Levine Phenotypic Age, computed from routine blood tests in EHRs, offers a feasible clinical tool for aging-related chemotoxicity risk stratification. Validation in diverse cohorts and the development of predictive models are needed.

## Simple Summary

Chemotherapy can cause adverse chemotoxicity, and some patients with colorectal cancer are more vulnerable than others. We aimed to determine whether a person’s biological age—how old their body appears based on routine blood tests, rather than their actual age—can help predict who is at higher risk for chemotoxicity. Using blood test data from 1129 colorectal cancer patients, we examined changes in biological aging over six months of chemotherapy, identified its risk factors, and explored how it relates to chemotoxicity. We found that chemotherapy tends to accelerate biological aging, and those with greater age acceleration were more likely to experience chemotoxicity. Accelerated biological aging over time was observed in both younger and older adults and was influenced by social factors, including area-level disparities. Our findings suggest that biological age could serve as a useful tool to identify high-risk patients and support more personalized cancer treatment planning.

## 1. Introduction

Colorectal cancer (CRC) remains a significant public health burden in the United States, being the third most commonly diagnosed cancer and the second leading cause of cancer-related deaths [1]. While advancements in screening, advanced treatment modalities, and systemic therapies have improved survival rates, leading to a growing population of CRC survivors, these individuals often face substantial long-term challenges due to chemotoxicity and related side effects [1]. Over the past two decades, the 5-year survival rate for CRC has increased, with approximately 65% of patients surviving beyond this milestone [2]. This rise in survivorship is attributed to earlier detection through colonoscopy screening and more effective treatment regimens, including the first-line chemotherapy (i.e., 5-Fluorouracil [5-FU]-based chemotherapy) [3]. However, a critical consequence of improved survival is the increasing number of patients experiencing persistent chemotoxicity and adverse symptoms following treatment.

A major challenge for CRC survivors is the substantial burden of chemotherapy-induced side effects, with approximately 50% of patients reporting such effects. These toxicities typically emerge during the first six months of first-line treatment and commonly include gastrointestinal (GI) complications and hematological disorders [4]. These adverse effects not only impair quality of life but may also result in early treatment discontinuation, emergency room visits, hospitalizations, dose reductions, cancer-related mortality, disease recurrence, and long-term functional impairments [4,5,6]. Current evidence indicates that between 40% and 70% of CRC survivors continue to experience persistent chemotherapy-related toxicity even after treatment completion, significantly compromising their daily functioning and overall well-being [6].

The growing population of CRC survivors and the high prevalence of treatment-related morbidity underscore the urgent need to further understand the mechanisms underlying chemotoxicity and develop targeted interventions to alleviate these complications [7,8]. Sensitive biomarkers of chemotoxicity remain limited due to inconclusive findings (e.g., variations by cancer stage), non-modifiable factors (e.g., race), or factors difficult to modify at the individual level (e.g., socioeconomic status, geographic disparities) [9]. However, emerging evidence suggests that biological aging processes may contribute to chemotherapy-induced toxicity in cancer patients [9]. Specifically, several modifiable (e.g., lifestyles, stress, and pro-inflammatory status) and non-modifiable (e.g., race, area-level socioeconomic status) factors are shown to be associated with cancer health outcomes [4]. These risk factors are closely associated with measures of biological aging; thus, biological aging markers may potentially be used as a sensitive risk factor for chemotoxicity.

Cancer patients frequently exhibit accelerated biological aging, wherein their physiological state appears significantly older than their chronological age would suggest, compared to healthy individuals [10,11,12]. This phenomenon is more evident in cancer patients than healthy individuals, who may maintain robust physical and cognitive function well into older age [10,13]. The accelerated aging observed in cancer patients manifests through multiple clinical dimensions, including premature frailty, increased vulnerability to age-related diseases, and functional decline in daily activities [14,15]. These effects stem largely from the unintended consequences of cancer treatments, which can trigger fundamental aging processes at the cellular and molecular level [10,16,17]. For example, therapy-induced DNA damage and epigenetic alterations, such as aberrant DNA methylation patterns, further contribute to the accelerated aging phenotype by disrupting normal cellular function and gene regulation [18,19]. Epidemiological studies demonstrate substantially higher rates of frailty among cancer survivors compared to age-matched controls [20], and in patients with hematopoietic cell transplant recipients showing particularly pronounced effects [21]. The risk of developing secondary malignancies increases dramatically in survivors, with childhood cancer patients facing a 20% cumulative incidence of subsequent neoplasms over 30 years [22]. Cardiovascular disease, cognitive decline, and other age-related conditions also emerge earlier in cancer survivors [23,24]. To our knowledge, only a few studies in other cancer types [25,26,27] showed potential links between biological aging markers and overall chemotoxicity incidences and cancer survival. The link between biological aging markers and chemotoxicity in CRC is unknown.

Biological age represents an individual’s functional and physiological condition compared to their actual chronological age, serving as an indicator of the biological aging processes [28]. Recent advances in aging research have introduced multiple biomarker-based approaches to quantify biological aging more precisely. Among these, epigenetic clocks such as the Horvath, Hannum, and GrimAge models analyze DNA methylation patterns and exhibit robust correlations with lifespan and age-related health outcomes [28]. However, these epigenetic techniques typically involve complex laboratory analyses and substantial costs, making them impractical for widespread epidemiological research [29]. As an alternative, Levine Phenotypic Age offers a more practical solution by combining routine peripheral blood tests—including albumin, creatinine, glucose, hepatic renal functions, and C-reactive protein levels using routine circulatory blood samples—to evaluate biological aging comprehensively, without requiring further blood assays [30]. This approach proves particularly valuable for large-scale studies due to its cost-effectiveness and accessibility. Levine Phenotypic Age acceleration quantifies the difference between an individual’s biological and chronological ages, where positive values suggest age acceleration [30]. The Levine Phenotypic Age has been associated with all-cause mortality and morbidity in the general population [30]. Though not originally designed for oncology, its use can be expanded to cancer populations due to its strong predictive value for several adverse health outcomes [28,30]. In CRC, chemotoxicities are common and influenced by physiological reserve, which is not fully captured by traditional clinical measures. Biological aging metrics like Levine Phenotypic Age, derived from inflammatory, metabolic, and organ function markers, may offer additional insight into treatment tolerance and toxicity risk [30]. Emerging research supports the link between accelerated biological aging and poor outcomes in cancer, including increased symptom burden, functional decline, and mortality [14,15]. However, the utility of Levine Phenotypic Age for measuring longitudinal changes in biological aging (both absolute values and age acceleration) during chemotherapy remains unexplored, along with the associated risk factors and their impact on chemotoxicity in CRC patients receiving 5-FU-based chemotherapy.

To address this knowledge gap, this study utilized real-world electronic health record (EHR) data from The Ohio State University Comprehensive Cancer Center to (1) assess the change in biological aging (i.e., raw biological age and age acceleration) as measured by Levine Phenotypic Age (without requiring further blood assays) over six months of 5-FU-based chemotherapy, (2) identify risk factors for biological aging, and (3) evaluate associations between biological aging (at baseline and over time) and chemotoxicity in adult patients with CRC. We hypothesized that elevated biological age and age acceleration at baseline (i.e., within 30 days before chemotherapy initiation) and increases in these measures during six months of chemotherapy would be associated with a higher risk of chemotherapy-related toxicity. While this hypothesis is exploratory, our hypothesis is informed by the conceptual link between physiological vulnerability captured by biological aging and chemotoxicity in cancer patients. Therefore, this study represents an initial investigation using real-world EHR data and should be interpreted as hypothesis-generating. The findings may help guide future work aimed at developing personalized tools for toxicity risk screening in cancer patients.

## 2. Materials and Methods

### 2.1. Data Collection

Our study employed a retrospective cohort design using de-identified EHR data from The Ohio State University Comprehensive Cancer Center, a major academic medical institution serving Ohio and surrounding Midwestern regions. Data spanned January 2013 through December 2019, with patient identification and data extraction performed by the institution’s Honest Broker Operations Committee in full compliance with HIPAA regulations. The analysis focused on 1129 adult patients (age ≥ 18 years) with stage II-III CRC who completed 8–12 cycles of 5-FU-based chemotherapy (e.g., 5-FU monotherapy, FOLFOX regimens, etc.) following tumor resection without stoma. Inclusion criteria included patients with a single primary CRC diagnosis, available routine blood test results at baseline (<30 days before starting chemotherapy) and 6-month post-treatment initiation, documented sociodemographic characteristics, and residential zip code information. Exclusion criteria included patients with active ostomies, chronic GI conditions, steroid/immunosuppressant use, a history of neoadjuvant therapy, and/or concurrent radiation/immunotherapy, cancer metastasis, pregnancy status, or incomplete medical records at either baseline or 6-month follow-up.

### 2.2. Measurements

#### 2.2.1. Patient Characteristics

The dataset incorporated comprehensive demographic (race, ethnicity, age, sex, marital status) and clinical variables (cancer sites, types of chemotherapy, history of other cancer treatments, cancer stages, body mass index, comorbidities, and lifestyle factors).

#### 2.2.2. Biological Age Assessment

Biological aging in this study was measured using the Levine Phenotypic Age. We computed the raw Levine Phenotypic Age using the formula provided below, as well as age acceleration, which represents whether a participant is biologically older or younger than expected for their chronological age. Levine Phenotypic Age formula [30] was implemented using nine routinely measured biomarkers: albumin, alkaline phosphatase, creatinine, glucose, C-reactive protein, lymphocyte percentage, mean cell volume, red cell distribution width, and white blood cell count at baseline (<30 days before starting chemotherapy) and 6 months after chemotherapy initiation.

*The calculation formula for Levine Phenotypic Age:*

$$Levine\;Phenotypic\;Age=141.50+\frac{Ln[-0.00553\times Ln(exp(\frac{-1.51714\;\times \;exp\left(xb\right)}{0.0076927}))]}{0.09165}$$

where xb = − 19.907 − 0.0336 × albumin (g/L) + 0.0095 × creatinine (μmol/L) + 0.1953 × glucose (mmol/L) + 0.0954 × LnCRP (mg/dL) − 0.0120 × lymphocyte percent (%) + 0.0268 × mean cell volume (fL) + 0.3306 × red cell distribution width (%) + 0.00188 × alkaline phosphatase (U/L) + 0.0554 × white blood cell count (1000 cells/μL) + 0.0804 × chronological age (years).

*Biological Age Acceleration:* We computed the biological age acceleration as Levine Phenotypic Age–Chronological Age at the time of biomarker collection [30].

Then, we analyzed raw Levine Phenotypic Age and age acceleration separately in our statistical models to assess their distinct relationships with chemotoxicity outcomes.

#### 2.2.3. Social Determinants of Health (SDOH)

We included available SDOH in the EHR data, including insurance type, marital status and employment information. Geographic disparities were quantified using the Area Deprivation Index (ADI) [31,32], a composite metric (scale 1–100) incorporating 17 neighborhood-level socioeconomic indicators. Patient zip codes were matched to 2015 ADI data, with scores categorized into tertiles denoting relative disadvantages.

#### 2.2.4. Chemotherapy Toxicity Event

*Global*, *GI*, *and Hematological toxicities:* Treatment-related adverse events were tracked for six months post-chemotherapy initiation. Chemotoxicity events were identified using clinician-documented International Classification of Diseases (ICD)-9 and ICD-10 diagnostic codes extracted from the EHR. These codes reflected treatment-related adverse events identified during routine clinical care. For this study, ICD-9/10 codes served as the sole source of chemotoxicity classification. Providers assigned ICD codes based either on the Common Terminology Criteria for Adverse Events (CTCAE v5.0) grading scale (which has high reliability = 0.95, and validity [33]), or on clinical judgment informed by laboratory and clinical data. A positive chemotoxicity case was defined as the presence of at least one documented ICD code indicating toxicity during the 6-month chemotherapy period [33].

### 2.3. Statistical Methods

Descriptive statistics characterized the study population. We utilized IBM SPSS Statistics (version 28.0; IBM Corp., Armonk, NY, USA). Descriptive statistics were computed for all variables, including means ± standard deviations or standard errors for continuous variables and frequencies (percentages) for categorical variables. First, we examined changes in biological aging (“original values of biological age” and “age acceleration: biological age–chronological age”) from baseline to 6 months after chemotherapy initiation, using the paired *t*-tests. Second, we examined the associations of potential risk factors with biological aging at baseline, 6 months after chemotherapy initiation, and changes in biological aging over time for 6 months, and also examined the associations of biological aging measures with chemotoxicity using a one-way analysis of variance (ANOVA). All ANOVA models met the assumptions of normality and homogeneity of variance. Lastly, we performed both unadjusted and adjusted logistic regression analyses (with Odds Ratio [OR] and 95% Confidence Interval [CI]) to examine the impact of biological aging measures on chemotoxicity outcomes. Adjusted models controlled for covariates and other significant risk factors of chemotoxicity. In our study, we identified covariates based on the literature and univariate analyses, which revealed some relationships with biological aging and chemotoxicity outcomes. These covariates included age groups, employment status, cancer stages, chemotherapy regimens, comorbidities, marital status, and ADI measured at baseline. Given our aim to assess biological aging as a general predictor of chemotherapy-related complications, we included all patients who received common 5-FU-based regimens, including FOLFIRI. Although Irinotecan in the FOLFIRI regimen is known to increase the risk of GI toxicity (e.g., diarrhea), this inclusion reflects real-world treatment heterogeneity and allows for broader assessment of phenotypic aging as a vulnerability marker. The treatment regimen was included as a covariate in all adjusted models. The variance inflation factor (VIF) among independent variables ranged from 1.1 to 3.7, indicating no multicollinearity in our analyses. Analyses were conducted using SPSS Version 28 (Chicago, IL, USA) with a two-sided significance level of 0.05. Multiple comparison corrections were not applied as our study was a hypothesis-driven study [34]. Missing data were minimal (<5% for all included variables). Therefore, we used complete case analysis to preserve internal validity.

## 3. Results

### 3.1. Participants Characteristics

Table 1 provides a summary of study participant characteristics, biological aging and chemotoxicity data in our study. The study population consisted of 1129 CRC patients with a mean chronological age of 57.3 years (standard deviation 13.7, range 18–89). The cohort comprised 53.4% female participants, with racial/ethnic distribution showing 68.1% Non-Hispanic White, 29.0% Non-Hispanic Black, and 2.9% other racial/ethnic groups. No Hispanic patients were identified in our dataset. A total of 55.8% of patients had colon cancer only, 34.5% had rectal cancer only, and 9.7% had cancer involving both sites. The majority (62.2%) were stage II, while 37.8% were stage III. Nearly 60% (59.7%) had a modified comorbidity index score above 2, indicating frequent comorbidities. The majority received GI surgery (79.7%), radiation (17.2%), and immunotherapy (5.1%). Health behaviors included 17.1% current smokers, 21.6% heavy alcohol users, and 42.9% reporting regular physical activity. Insurance coverage showed 61.5% with private insurance and 38.5% with Medicare/Medicaid. A total of 56.0% of patients were married/partnered, and 44.0% were divorced/widowed/single. The majority of participants (42.0%) lived in moderately disadvantaged areas (ADI 34–66), while 33.0% resided in the least disadvantaged neighborhoods (ADI 0–33) and 25.0% in the most disadvantaged (ADI 67–100). Nearly half (47.1%) were currently employed, 27.6% were unemployed, and a quarter (25.4%) were retired.

*Biological aging*: The raw biological age, as measured by raw Levine Phenotypic Age, showed an average increase from 59.1 years before chemotherapy to 61.8 years after six months of treatment, representing a mean increase of 2.7 years (Table 1). The age acceleration (i.e., difference between biological and chronological age) widened from 1.2 years at baseline to 2.8 years post-treatment, indicating accelerated aging worsened during chemotherapy.

*Chemotoxicity:* Chemotoxicity was frequently observed, with 56.3% experiencing global chemotoxicity, 40.8% GI toxicity, and 22.8% hematological toxicity. These findings collectively suggest significant biological aging effects and substantial treatment-related morbidity in this colorectal cancer population undergoing chemotherapy.

### 3.2. Risk Factors of Raw Levine Phenotypic Age

Table 2 describes the risk factors of biological aging as measured by raw Levine Phenotypic Age, at baseline, 6 months, and changes from baseline to 6 months. As expected, younger patients (age < 50) showed significantly lower Levine Phenotypic Age at baseline (48.7 vs. 69.9, *p* < 0.001) and 6 months (51.7 vs. 72.3, *p* < 0.001). Notably, stage II patients experienced a greater Phenotypic Age increase over time as compared to stage III patients (2.3 vs. 1.8 years, *p* = 0.013). Patients with higher comorbidity burdens showed greater Levine Phenotypic Age increases over time (3.0 vs. 2.4 years, *p* = 0.013). Socioeconomic factors significantly impacted aging trajectories: divorced/widowed/single, residents of disadvantaged neighborhoods (ADI 67–100), and unemployed/retired study participants, all demonstrated accelerated biological aging. These findings highlight how clinical and socioeconomic factors distinctly influence biological aging during cancer treatment.

### 3.3. Risk Factors of Age Acceleration

Table 3 provides the risk factors for age acceleration (the age gap between Levine Phenotypic Age and chronological age during chemotherapy). The younger adult group showed greater age acceleration compared to the older adult group (with differences of 2.3 vs. 0.9 years at baseline and 4.1 vs. 2.3 years after treatment, Ps < 0.001). Both age groups showed similar increases in age acceleration over the treatment period, with younger patients gaining 1.8 years and older patients gaining 1.4 years. Clinical characteristics showed important associations with age acceleration. Patients with stage III cancer presented with lower baseline age acceleration than stage II patients (1.3 vs. 1.9 years, *p* < 0.001) and experienced more pronounced increases in aging during treatment (3.8 vs. 2.6 years, *p* < 0.001). Patients with more comorbidities experienced a more substantial increase in age acceleration during chemotherapy (1.8 vs. 1.4 years, *p* < 0.001). Socioeconomic factors revealed disparities in age acceleration. Unmarried patients maintained higher age acceleration values than their married counterparts at both baseline (2.2 vs. 1.0 years, *p* = 0.006) and post-treatment (3.8 vs. 2.6 years, *p* < 0.001). Neighborhood disadvantages showed strong associations with age acceleration, as residents of the most disadvantaged areas (ADI: 67–100) had higher age acceleration values of 2.1 years at baseline and 4.1 years post-treatment, compared to residents of the least disadvantaged areas (*p* = 0.008 and *p* < 0.001, respectively). Employment status significantly influenced age acceleration over time, with unemployed or retired patients experiencing greater increases than employed patients (1.7 vs. 1.5 years, *p* < 0.001).

### 3.4. Associations of Biological Age with Chemotoxicity

Table 4 shows the results of associations of biological aging (baseline and changes over time) with chemotoxicity outcomes. Patients who experienced global chemotoxicity showed markedly higher raw Levine Phenotypic Age compared to those without toxicity, with values of 60.5 versus 57.7 years at baseline (*p* < 0.001) and 63.8 versus 59.8 years after treatment (*p* < 0.001). These patients also demonstrated greater increases in raw Levine Phenotypic Age over time (3.3 vs. 2.1 years, *p* < 0.001). Patients reported GI and hematological toxicities had higher baseline raw Levine Phenotypic Age, and greater increases in raw Levine Phenotypic Age over time, as compared to the patients without these adverse events.

Similar patterns emerged for age acceleration, where global chemotoxicity cases had substantially higher age acceleration at baseline (2.1 vs. 0.3 years, *p* < 0.001), post-treatment (4.3 vs. 1.3 years, *p* < 0.001), and over time (2.2 vs. 1.1 years, *p* < 0.001). Similar results were found in GI and hematological toxicities. Despite this, age acceleration rates (i.e., age acceleration changes over time) were similar among groups with GI (1.9 years accelerated) and hematological toxicities (1.9 years accelerated). The age acceleration differences were even more pronounced, with hematological toxicity cases showing higher baseline (2.3 vs. 0.1 years, *p* < 0.001) and post-treatment (4.2 vs. 1.4 years, *p* < 0.001) values than those without hematological toxicity.

### 3.5. Impact of Biological Age on Chemotoxicity

Given the significant relationship between biological age and chemotoxicity outcomes shown in Table 4, we performed further logistic regression analyses to examine the impact of biological age on chemotoxicity (i.e., strengths of the associations) in Table 5. The analysis revealed significant associations between biological aging measures and chemotoxicity risk in both unadjusted and adjusted models. For global chemotoxicity, baseline Levine Phenotypic Age showed strong predictive value with an adjusted odds ratio (aOR) of 1.27 (95% CI: 1.22–1.32, *p* < 0.001), indicating a 27% increased risk per year of higher biological age. Changes in Phenotypic Age over time demonstrated an even greater association with risk (aOR = 2.74, 95% CI: 2.45–3.04, *p* < 0.001). Similar patterns emerged for age acceleration measures, with baseline values (aOR = 1.27, *p* < 0.001) and changes over time (aOR = 2.74, *p* < 0.001) both showing significant links to global toxicity risk.

For GI and hematological toxicities, results were similar showing significant associations between biological measures and chemotoxicity. Overall, compared to Levine Phenotypic Age and its age acceleration at baseline, changes in these biological aging measures were more strongly associated with chemotoxicity outcomes. Most of the biological aging measures at baseline and changes over time (Levine Phenotypic Age and age acceleration) in unadjusted models reached significance in the adjusted models.

## 4. Discussion

This study provides new evidence that chemotherapy accelerates biological aging in CRC patients, as measured by Levine Phenotypic Age derived from routinely available EHR data. Our findings suggest that biological aging progresses significantly over six months of treatment, with Phenotypic Age increasing by an average of 2.7 years, while age acceleration increased from 1.2 to 2.8 years. Our study also demonstrates that biological aging during chemotherapy is influenced by a combination of clinical factors, including comorbidity burden, along with socioeconomic determinants such as marital status, neighborhood characteristics, and employment status. The findings identify specific patient subgroups who may be particularly vulnerable to accelerated biological aging during cancer treatment, suggesting potential targets for more personalized treatment approaches and supportive care interventions. Below, we interpret our findings in the context of prior research, discuss clinical implications, and explore potential biological mechanisms.

### 4.1. Biological Aging During Chemotherapy

Our study provides evidence that 5-FU-based chemotherapy accelerates biological aging in CRC patients, aligning with findings from breast cancer research and preclinical models [9,35]. Although cancer therapies—including chemotherapy, radiotherapy, and immunotherapy—are designed to induce apoptosis or senescence in malignant cells, they also inadvertently promote senescence in healthy tissues, contributing to systemic biological aging [9,35]. Chemotherapy-induced biological aging likely arises through multiple mechanisms such as DNA damage, cellular senescence, and chronic inflammation [9,12,26,28,36,37]. First, chemotherapy agents cause DNA damage and cellular senescence, as demonstrated by elevated expression of senescence markers such as p16^INK4A^ in breast cancer patients following treatment [38]. Second, chemotherapy is associated with epigenetic aging and telomere attrition. For instance, large-scale studies reveal that breast cancer survivors treated with chemotherapy exhibit 1–2 years of accelerated epigenetic aging (measured by the Horvath and GrimAge clocks) compared to non-chemotherapy controls [36]. Additionally, chemotherapy patients showed increased inflammatory markers (e.g., IL-6, CRP) in CRC [39], which correlate with reduced survival and worse cancer health outcomes. Lastly, agents such as 5-FU and oxaliplatin generate reactive oxygen species (ROS), overwhelming endogenous antioxidant defenses and accelerating cellular aging [40]. Our study builds on this literature by showing that Levine Phenotypic Age—a composite biomarker of complete blood counts, inflammation, and hepatorenal function—can capture these aging-related mechanisms in CRC. Chemotherapy induces a senescence-associated secretory phenotype (SASP)**,** leading to the release of pro-inflammatory cytokines (e.g., IL-6, CRP) that exacerbate tissue damage and systemic aging [41]. Patients with elevated baseline Levine Phenotypic Age may have diminished stress response capacity, rendering them more vulnerable to treatment toxicity [42]. The integration of biological aging biomarkers, such as Levine Phenotypic Age, into clinical practice could improve risk stratification and guide therapeutic decisions.

### 4.2. Risk Factors for Biological Aging During Chemotherapy

In addition to the aging effects of chemotherapy, we extend this by identifying key risk factors for biological aging. The elevated biological aging in stage III versus stage II patients in our study may reflect combined effects of more aggressive therapies (e.g., chemotherapy-induced senescence) and greater tumor burden (e.g., pro-inflammatory signaling), both known to accelerate aging [4,43]. In our study, older adults exhibited higher baseline biological age, while younger adults showed greater age acceleration. This could be possibly due to stronger treatments [44] and chronic exposure to stress, unhealthy lifestyles, and reduced resilience [45]. This highlights that younger individuals are also vulnerable to chemotherapy-induced aging. While older adults’ aging likely reflects cumulative inflammation and chronic disease, younger adults may experience accelerated aging due to acute stressors and intensive therapies. Biological aging and age acceleration were also associated with comorbidities, higher area-level deprivation, unemployment, and unmarried status. Social disadvantages, loneliness, and poor living conditions likely contribute to stress-related biological dysregulation [46]. These findings underscore the need for age- and context-sensitive strategies—such as managing comorbidities and providing socioeconomic support—to reduce aging-related chemotherapy risks.

### 4.3. Biological Age Is a Risk Factor for Chemotoxicity

Our study is the first to examine associations between biological aging and 5-FU-based chemotoxicity outcomes in CRC using Levine Phenotypic Age. Patients with higher baseline Phenotypic Age and greater increases over time had elevated risk of global, GI, and hematological toxicities. Several studies support our findings showing associations between biological aging and chemotoxicity in other cancer types [47,48,49]. In patients with breast cancer, T cell p16^INK4a^ expression was positively associated with peripheral neuropathy after 1.5 years of treatment with paclitaxel and docetaxel [50], and fatigue after one month of taxane-based chemotherapy [51]. Multiple studies have shown a link between baseline telomere length and chemotherapy-induced toxicities in breast cancer and lymphoma patients [47,48,49]. Additionally, recent research identified a connection between p16^INK4a^ expression and epigenetic aging markers such as the Hannum and PhenoAge DNA methylation clocks, along with various mRNA signatures of T cell senescence. This study suggests that aging-related biomarkers, particularly p16^INK4a^ and epigenetic clocks, may help predict physical and cognitive frailty in older adults with blood cancers [52]. Despite the current literature on biological aging and chemotoxicity mentioned above, indicators of epigenetic age (e.g., DNA methylation clocks) and T cell expression profiles remain limited for routine clinical use due to insufficient clinical validation, high costs, and the technical complexity of measuring these markers [9]. Our use of an accessible, EHR-derived biological age marker, ‘Levine Phenotypic Age’, offers a pragmatic and scalable method for identifying and monitoring high-risk CRC patients before and during chemotherapy.

### 4.4. Clinical Implications

Our findings suggest that biological aging assessments could improve risk stratification before chemotherapy. However, it is also possible that biological aging reflects not only baseline vulnerability, but also physiological changes triggered by chemotherapy-related stress or toxicity. Although Levine Phenotypic Age was originally designed to predict mortality, its association with treatment toxicity in this context may indicate that it captures underlying biological frailty that is further revealed or amplified during chemotherapy. Thus, rather than serving solely as a pre-treatment risk factor, Levine Phenotypic Age may function as a complementary marker of the aging-related physiological response to treatment. Furthermore, researchers can consider developing risk screening tools by integrating biological aging markers and relevant clinical and social factors to guide treatment decisions. Second, patients with high Levine Phenotypic Age and age acceleration may benefit from dose modifications, comorbidity management, enhanced supportive care, or anti-aging interventions such as antioxidants (e.g., coenzyme Q10) or lifestyle interventions to mitigate toxicity [53].

### 4.5. Limitations and Future Directions

This study has several limitations. As a retrospective secondary data analysis, true causal inferences between biological aging and chemotoxicity are limited. Important individual-level variables—such as education, income, stress levels, and diet quality—were unavailable and may confound observed associations. Additionally, reliance on EHR data and clinician-documented ICD codes, without routine use of standardized CTCAE grading or patient-reported outcomes, may result in underreporting of chemotoxicity. The use of only two follow-up timepoints (baseline and 6 months) restricts insight into the dynamic trajectory of biological aging and its longer-term impact on survivorship outcomes such as frailty, function, fatigue, quality of life, and secondary cancers. We adjusted for regimen type in our models. Nonetheless, we acknowledge that agent-specific toxicities may partially influence these outcomes, and future prospective studies could explore biological aging’s predictive value within specific chemotherapy regimens. Moreover, this study was conducted at a single academic cancer center, potentially limiting generalizability to broader and more diverse populations. Future studies should validate Levine Phenotypic Age in larger, more diverse CRC cohorts, employ both clinician- and patient-reported toxicity assessments, and compare aging metrics—including molecular and clinical frailty indices—to optimize aging-informed risk prediction and management strategies across CRC populations.

## 5. Conclusions

This study suggests that chemotherapy accelerates biological aging in CRC patients. Levine’s Phenotypic Age, calculated using routine blood test results from EHR, served as a potential predictor of chemotoxicity. Both young and older adults, those with greater comorbidities, more advanced cancer stages, and socioeconomic disadvantages (including area-level disparities), are at higher risk for chemotoxicity. However, given the retrospective and observational nature of the analysis, causality cannot be inferred. It is also important to consider the reverse hypothesis—that chemotherapy-related toxicity may itself contribute to biological age acceleration or a worsening Levine score, which could, in turn, be associated with increased mortality risk. These findings should be interpreted as hypothesis-generating and warrant further validation in prospective studies. Future research should validate the current findings in different cohorts of patients with CRC to facilitate the use of blood-based biological aging markers in routine clinical use, develop aging-based chemotoxicity screening tools, and explore whether targeting aging mechanisms (e.g., anti-inflammatories) can improve treatment tolerance and survivorship outcomes.

## Figures and Tables

**Table 1:** Characteristics of Participants.

Demographic Factors	Total Samples (N = 1129)
Age (years), mean ± SD (range)	57.3 ± 13.7 (18–89)
Female (*n*, %)	603 (53.4)
Race/Ethnicity (*n*, %): Non-Hispanic White	769 (68.1)
Non-Hispanic Black	327 (29.0)
Non-Hispanic Other	33 (2.9)
**Clinical Factors (*n*, %)**	
Cancer Site: Colon only	630 (55.8)
Rectal only	389 (34.5)
Colon and Rectal	110 (9.7)
Cancer stages II *	702 (62.2) *
III	427 (37.8)
5-FU (fluorouracil)-based chemotherapy regimens with an average of 2600 mg/m^2^ and 10 cycles	
FOLFOX (infusion)	459 (40.6)
FOLFIRI (infusion)	325 (28.8)
CAPEOX (oral)	199 (17.6)
Single-Agent 5-FU (infusion)	146 (13.0)
Body mass index (BMI): Obese (≥30)	226 (20.0)
Overweight (25≤, <30)	452 (40.0)
Normal (21≤, <25)	395 (35.0)
Underweight (<21)	56 (5.0)
Modified Comorbidity Index (≥2)	1035 (59.7)
Previous cancer treatment history:	
Radiation	172 (17.2)
Immunotherapy	58 (5.1)
GI surgery	881 (79.7)
Cancer Health Behaviors (Yes):	
Current Smoking Status	193 (17.1)
Current Heavy Alcohol Use	244 (21.6)
Regular Physical Activity	482 (42.9)
**Social Determinants of Health (SDOH) (*n*, %)**	
Primary Insurance Types: Private	694 (61.5)
Medicare/Medicaid	435 (38.5)
Marital status: Married/Partnered	632 (56.0)
Divorced/Widowed/Single	497 (44.0)
Area Deprivation Index (ADI), Tertile: 0–33	372 (33.0)
34–66	470 (42.0)
67–100	287 (25.0)
Employment Status: Employed	531 (47.1)
Unemployed	311 (27.6)
Retired	287 (25.4)
**Biological Aging**	
**Raw Levine Phenotypic Age, mean ± SD (range)**	
at T0 (before chemotherapy)	59.1 ± 13.8 (19–95)
at T1 (6 months after chemotherapy)	61.8 ± 14.1 (19–96)
mean changes in biological age over time from T0 to T1	2.7 ± 1.2 (0.0–2.1)
**Biological Age Acceleration**	
(Differences from Levine Phenotypic Age to Chronological Age), mean ± SD	
at T0 (before chemotherapy)	1.2 ± 0.5
at T1 (6 months after chemotherapy)	2.8 ± 0.7
mean changes in biological age acceleration over time from T0 to T1	1.6 ± 0.5
**Chemotoxicity Incidences for 6 months of chemotherapy (*n*, %)**	
Clinician-Reported Global Chemotoxicity	636 (56.3)
Clinician-Reported Gastrointestinal Chemotoxicity	460 (40.8)
Clinician-Reported Hematological Chemotoxicity	258 (22.8)

Note: SD = standard deviation. * Non-metastatic, high-risk stage II CRC (*n* = 594, 84.6% among 702 patients with stage II CRC).

**Table 2:** Factors related to the Raw Levine Phenotypic Age Over the Course of Chemotherapy.

	Raw Levine Phenotypic Age Mean ± SE)						
	At Baseline	F, *p* ^a^	6 Months After Chemotherapy ^c^	F, *p* ^a^	Changes Overtime	Paired t, *p* ^b^ ^Time Effects^	F, *p* ^a^ ^Group Effects^
Age group:		38.6, **<0.001**		38.8, **<0.001**			7.98, **0.005**
Young Adults (18 ≤ age < 50, *n* = 332)	48.7 (0.4)		51.7 (0.4)		3.0 (0.06)	28.75, **<0.001**	
Older Adults (age ≥ 50, *n* = 797)	69.9 (0.9)		72.3 (0.3)		2.4 (0.05)	47.94, **<0.001**	
Sex: Male	58.2 (0.4)	3.35, 0.067	60.5 (0.4)	3.35, 0.067	2.3 (0.02)	35.43, **<0.001**	0.08, 0.769
Female	60.0 (0.3)		63.0 (0.5)		3.0 (0.03)	43.71, **<0.001**	
Race/Ethnicity:		2.97, 0.051		3.23, **0.040**			1.12, 0.328
Non-Hispanic White	60.1 (0.4)		63.3 (0.4)		3.2 (0.03)	52.01, **<0.001**	
Non-Hispanic Black	59.6 (0.3)		62.1 (0.5)		2.5 (0.02)	18.73, **<0.001**	
Non-Hispanic Other	57.5 (0.4)		56.9 (0.4)		2.4 (0.01)	8.25, **<0.001**	
Cancer Site: Colon only	58.0 (0.5)	2.31, 0.101	60.8 (0.5)	3.32, 0.112	2.8 (0.02)	10.39, 0.135	1.18, 0.313
Rectal only	60.0 (0.4)		62.7 (0.3)		2.7 (0.03)	13.44, 0.112	
Colon and Rectal	59.3 (0.3)		61.6 (0.6)		2.3 (0.02)	16.51, 0.101	
Cancer stages: II	58.5 (0.4)	1.58, 0.162	61.5 (0.5)	1.79, 0.112	3.0 (0.02)	21.85, **<0.001**	4.35, **0.013**
III	59.7 (0.5)		62.1 (0.6)		2.4 (0.03)	49.33, **<0.001**	
5-FU (fluorouracil)-based chemotherapy		1.11, 0.523		0.91, 0.941			1.18, 0.313
FOLFOX (infusion)	57.6 (0.4)		60.6 (0.5)		3.0 (0.4)	5.6, 0.542	
FOLFIRI (infusion)	58.4 (0.9)		61.0 (0.8)		2.6 (0.8)	6.3, 0.481	
CAPEOX (oral)	60.8 (1.3)		63.2 (1.5)		2.4 (1.3)	2.1, 0.994	
Single-Agent 5-FU (infusion)	58.6 (1.7)		61.2 (1.8)		2.6 (1.5)	6.1, 0.501	
Body Mass Index (BMI)		1.65, 0.209		1.11, 0.123			2.01, 0.129
Obese (≥30)	58.8 (0.9)		61.8 (0.9)		3.0 (0.9)	5.4, 0.499	
Overweight (≥25, <30)	59.4 (0.8)		62.0 (0.8)		2.6 (0.8)	6.1, 0.312	
Normal (≥21, <25)	59.1 (0.6)		61.7 (0.6)		2.6 (0.5)	5.5, 0.561	
Underweight (<21)	59.1 (1.3)		61.7 (1.3)		2.6 (1.2)	6.5, 0.209	
Modified Comorbidity Index: ≥2	58.9 (0.4)	1.61, 0.203	61.7 (0.4)	3.08, 0.080	2.9 (0.04)	40.23, **<0.001**	20.3, **<0.001**
<2	58.1 (0.5)		60.6 (0.5)		2.5 (0.04)	39.15, **<0.001**	
History of Radiation: Yes	58.5 (0.4)	1.77, 0.233	61.2 (0.5)	1.66, 0.129	2.7 (0.5)	9.15, 0.121	1.87, 0.133
No	59.7 (0.6)		62.5 (0.5)		2.8 (0.4)	5.55, 0.312	
History of Immunotherapy: Yes	58.1 (0.4)	1.56, 0.122	60.8 (0.4)	1.82, 0.132	2.7 (0.02)	14.23, 0.121	2.01, 0.122
No	60.1 (0.5)		62.8 (0.6)		2.7 (0.03)	11.15, 0.212	
History of GI surgery: Yes	58.0 (0.5)	1.89, 0.132	60.7 (0.5)	1.55, 0.122	2.7 (0.03)	39.45, **<0.001**	2.22, 0.159
No	60.2 (0.6)		62.9 (0.5)		2.7 (0.04)	29.15, **<0.001**	
Current Smoking Status: Yes	58.9 (0.4)	1.96, 0.195	61.3 (0.5)	2.71, 0.231	2.4 (0.04)	51.45, **<0.001**	2.56, 0.222
No	59.3 (0.5)		62.3 (0.4)		3.0 (0.01)	43.21, **<0.001**	
Current Heavy Alcohol Use: Yes	58.5 (0.5)	1.75, 0.133	61.1 (0.4)	1.88, 0.195	2.6 (0.05)	49.31, **<0.001**	2.11, 0.298
No	59.7 (0.6)		62.6 (0.5)		2.9 (0.04)	41.21, **<0.001**	
Routine Physical Activity: Yes	57.8 (0.5)	1.79, 0.195	60.2 (0.4)	2.09, 0.185	2.4 (0.05)	51.41, **<0.001**	2.41, 0.187
No	60.4 (0.5)		63.4 (0.4)		3.0 (0.04)	39.41, **0.004**	
Primary Insurance Types: Private	58.9 (0.4)	1.72, 0.194	61.3 (0.5)	2.11, 0.132	2.4 (0.4)	12.41, 0.496	2.41, 0.195
Medicare/Medicaid	59.3 (0.5)		62.5 (0.4)		3.2 (0.5)	19.31, 0.312	
Marital status: Married/Partnered	58.5 (0.5)	1.74, 0.175	60.0 (0.5)	2.21, 0.110	2.5 (0.05)	44.07, **<0.001**	8.9 **<0.001**
Divorced/Widowed/Single	59.7 (0.5)		62.8 (0.5)		2.8 (0.05)	36.91, **<0.001**	
ADI, Tertile: 0–33	58.2 (0.7)	1.98, 0.138	60.7 (0.7)	3.19, **0.041**	2.5 (0.08)	22.8, **<0.001**	12.99, **<0.001**
34–66	58.7 (0.6)		61.4 (0.5)		2.7 (0.06)	32.46, **<0.001**	
67–100	60.4 (0.5)		63.4 (0.3)		3.0 (0.06)	40.21, **<0.001**	
Employment Status: Employed	57.6 (0.5)	7.21, **<0.001**	60.4 (0.5)	7.60, **<0.001**	2.6 (0.05)	35.12, **<0.001**	4.88, **<0.001**
Unemployed/Retired	60.6 (0.7)		63.4 (0.7)		2.8 (0.07)	28.36, **<0.001**	

Note: Characteristics of variables were described using means with standard error (SE). *p*-values in bold if they are <0.05, as this is considered the statistical significance level, based on using ^a^ ANOVA for group differences. F statistic: A ratio used in ANOVA to test whether group means are significantly different. A larger F value suggests greater differences between group means. ^b^ paired *t*-tests for time effects. ^c^ Age acceleration at 6 months = Biological age at 6 months–(baseline chronological age + 0.5 years).

**Table 3:** Factors related to Biological Age Acceleration (i.e., Differences from Biological Age to Chronological Age) Over the course of Chemotherapy.

	Biological Age Acceleration (Mean ± SE)						
	At Baseline	F, *p* ^a^	6 Months After Chemotherapy ^c^	F, *p* ^a^	Changes Over Time	Paired t, *p* ^b^ ^Time Effects^	F, *p* ^a^ ^Group Effects^
Age group:		30.371, **<0.001**		19.558, **<0.001**			7.985, **0.005**
Young Adults (18 ≤ age < 50, *n* = 332)	2.3 (0.3)		4.1 (0.3)		1.8 (0.1)	38.71, **<0.001**	
Older Adults (age ≥ 50, *n* = 797)	0.9 (0.2)		2.3 (0.2)		1.4 (0.1)	45.51, **<0.001**	
Sex: Male	1.4 (0.2)	0.064, 0.800	3.0 (0.2)	0.098, 0.754	1.6 (0.1)	40.31, **0.001**	0.086, 0.769
Female	1.8 (0.2)		3.4 (0.2)		1.6 (0.1)	39.51, **0.003**	
Race/Ethnicity:		0.436, 0.647		0.785, 0.456			1.115, 0.328
Non-Hispanic White	1.7 (0.2)		3.5 (0.2)		1.8 (0.1)	49.65, **0.001**	
Non-Hispanic Black	1.3 (0.4)		2.7 (0.4)		1.4 (0.1)	51.55, **0.004**	
Non-Hispanic Other	1.8 (0.7)		3.4 (0.8)		1.6 (0.2)	48.43, **0.003**	
Cancer Site: Colon only	1.5 (0.2)	0.351, 0.641	2.8 (0.2)	0.841, 0.533	1.3 (0.3)	24.51, 0.121	0.549, 0.299
Rectal only	1.8 (0.2)		3.5 (0.3)		1.7 (0.3)	51.55, **0.006**	
Colon and Rectal	1.5 (0.1)		3.3 (0.5)		1.8 (0.3)	21.39, 0.149	
Cancer stages: II	1.9 (0.2)	4.958, **<0.001**	3.8 (0.4)	6.582, **<0.001**	1.9 (0.1)	49.55, **0.005**	4.281, **<0.001**
III	1.3 (0.3)		2.6 (0.3)		1.3 (0.8)	29.41, 0.134	
5-FU (fluorouracil)-based chemotherapy		1.312, 0.985		0.412, 0.512			0.102, 0.132
FOLFOX (infusion)	1.3 (0.5)		2.6 (0.5)		1.3 (0.5)	13.12, 0.156	
FOLFIRI (infusion)	1.5 (0.7)		3.1 (0.8)		1.6 (0.7)	14.61, 0.952	
CAPEOX (oral)	1.9 (0.9)		3.9 (0.9)		2.0 (0.9)	13.05, 0.121	
Single-Agent 5-FU (infusion)	1.7 (1.1)		3.3 (1.1)		1.9 (1.1)	19.11, 0.232	
Body Mass Index (BMI)		0.192, 0.542		0.293, 0.133			1.102, 0.432
Obese (≥30)	1.7 (0.6)		3.1 (0.6)		1.4(0.6)	5.6, 0.988	
Overweight (≥25, <30)	1.6 (0.7)		3.4 (0.6)		1.8 (0.6)	7.1, 0.999	
Normal (≥21, <25)	1.5 (0.4)		3.0 (0.5)		1.5 (0.4)	11.2, 0.516	
Underweight (<21)	1.6 (1.1)		3.3 (1.1)		1.7(1.1)	13.5, 0.309	
Modified Comorbidity Index: ≥2	1.3 (0.2)	0.025, 0.875	3.1 (0.2)	1.679, 0.195	1.8 (0.1)	39.41, **0.005**	20.33, **<0.001**
<2	1.9 (0.2)		3.3 (0.2)		1.4 (0.1)	45.44, **0.001**	
History of Radiation: Yes	1.1 (0.3)	0.412, 0.521	2.9 (0.4)	0.334, 0.563	1.8 (0.2)	40.55, **0.005**	0.007, 0.934
No	2.1 (0.1)		3.5 (0.2)		1.4 (0.1)	51.55, **0.007**	
History of Immunotherapy: Yes	1.8 (0.3)	5.078, **0.024**	3.6 (0.3)	0.385, 0.726	1.8 (0.1)	43.59, **0.001**	1.035, 0.309
No	1.4 (0.2)		2.8 (0.2)		1.4 (0.1)	45.61, **0.010**	
History of GI surgery: Yes	1.5 (0.2)	0.684, 0.408	3.3 (0.2)	0.699, 0.403	1.8 (0.1)	40.99, **0.005**	0.056, 0.812
No	1.7 (0.4)		3.1 (0.4)		1.4 (0.1)	25.61, 0.112	
Current Smoking Status: Yes	1.4 (0.1)	0.415, 0.122	2.8 (0.1)	0.423, 0.412	1.4(0.1)	59.55, **0.009**	0.322, 0.599
No	1.8 (0.2)		3.6 (0.1)		1.8(0.1)	61.15, **0.003**	
Current Heavy Alcohol Use: Yes	1.3 (0.1)	0.333, 0.233	2.5 (0.1)	0.012, 0.999	1.2 (0.1)	55.69, **0.010**	0.043, 0.891
No	1.5 (0.3)		2.9 (0.2)		1.4 (0.1)	63.52, **0.009**	
Routine Physical Activity: Yes	1.2 (0.1)	0.513, 0.431	2.8 (0.2)	0.333, 0.444	1.6 (0.1)	54.59, **<0.001**	0.019, 0.981
No	2.0 (0.2)		3.6 (0.1)		1.6 (0.1)	55.61, **0.010**	
Primary Insurance Types: Private	1.5 (0.2)	0.222, 0.132	3.3 (0.2)	0.122, 0.159	1.8 (0.1)	13.73, 0.232	0.233, 0.481
Medicare/Medicaid	1.7 (0.1)		3.1 (0.2)		1.4 (0.1)	19.65, 0.167	
Marital status: Married/Partnered	1.0 (0.2)	5.174, **0.006**	2.6 (0.2)	8.351, **<0.001**	1.6 (0.1)	65.05, **0.001**	8.984, **<0.001**
Divorced/Widowed/Single	2.2 (0.2)		3.8 (0.2)		1.6 (0.1)	49.10, **0.010**	
ADI, Tertile: 0–33	1.1 (0.3)	4.819, **0.008**	2.5 (0.1)	8.324, **<0.001**	1.4 (0.1)	49.89, **0.009**	12.993, **<0.001**
34–66	1.6 (0.2)		3.2 (0.2)		1.6 (0.1)	59.11, **0.012**	
67–100	2.1 (0.2)		4.1 (0.2)		2.0 (0.1)	48.51, **0.019**	
Employment Status: Employed	1.1 (0.2)	1.842, 0.118	2.8 (0.2)	2.319, 0.055	1.5 (0.1)	53.59, **0.013**	4.889, **<0.001**
Unemployed/Retired	1.7 (0.2)		3.5 (0.3)		1.7 (0.1)	47.51, **0.005**	

Note: Characteristics of variables were described using means with standard error (SE). *p*-values in bold if they are <0.05, as this is considered the statistical significance level, based on either using ^a^ ANOVA for between-group differences or ^b^ paired *t*-tests for within-group changes from baseline to 6 months post-chemotherapy. ^c^ Age acceleration at 6 months = Biological age at 6 months–(baseline chronological age + 0.5 years).

**Table 4:** Associations of Biological Aging with Chemotoxicity.

**Chemotoxicity**	**Raw Levine Phenotypic Age, mean ± SE**						
	**At** **Baseline**	**F, *p* ^a^**	**6 Months After Chemotherapy**	**F, *p* ^a^**	**Changes** **Overtime**	**Paired t, *p* ^b^** **^Time Effects^**	**F, *p* ^a^** **^Group Effects^**
Global. Yes	60.5 (0.4)	81.71, **<0.001**	63.8 (0.5)	129.01, **<0.001**	3.3 (0.04)	8.1, **0.005**	542.71, **<0.001**
No	57.7 (0.5)		59.8 (0.5)		2.1 (0.04)	69.5, **0.001**	
GI. Yes	60.6 (0.8)	7.53, **0.006**	62.8 (0.1)	8.53, **0.004**	2.2 (0.2)	33.5, **0.009**	107.52, 0.077
No	57.6 (0.4)		60.8 (0.1)		3.2 (0.4)	49.5, **0.010**	
Hematological. Yes	60.1 (0.6)	6.19, **0.013**	63.5 (0.7)	8.52, **0.004**	3.4 (0.7)	39.6, **0.015**	17.92, **<0.001**
No	58.1 (0.4)		60.1 (0.4)		2.0 (0.4)	40.5, **0.032**	
**Chemotoxicity**	**Age Acceleration (Differences from Levine Phenotypic Age to Chronological Age)**						
	**At** **Baseline**	**F, *p* ^a^**	**6 Months After Chemotherapy ^c^**	**F, *p* ^a^**	**Changes** **Overtime**	**Paired t, *p* ^b^** **^Time Effects^**	**F, *p* ^a^** **^Group Effects^**
Global. Yes	2.1 (0.2)	190.07, **<0.001**	4.3 (0.2)	373.40, **<0.001**	2.2 (0.1)	55.1, **0.009**	542.74, **<0.001**
No	0.3 (0.2)		1.3 (0.2)		1.1 (0.1)	59.3, **0.013**	
GI. Yes	1.5 (0.2)	3.13, **0.077**	3.4 (0.4)	5.78, **0.016**	1.9 (0.1)	53.1, **0.007**	8.53, **0.004**
No	0.9 (0.4)		2.2 (0.2)		1.3 (0.4)	49.5, **0.029**	
Hematological. Yes	2.3 (0.3)	27.81, **<0.001**	4.2 (0.3)	36.50, **<0.001**	1.9 (0.1)	66.3, **0.010**	17.87, **<0.001**
No	0.1 (0.2)		1.4 (0.2)		1.3 (0.1)	65.5, **0.005**	

Note: Characteristics of variables were described using means with standard error (SE). *p*-values in bold if they are <0.05, as this is considered the statistical significance level, based on either using ^a^ ANOVA for group differences or ^b^ paired *t*-tests for time effects. ^c^ Age acceleration = Biological age at 6 months–(chronological age + 0.5 years).

**Table 5:** Impact of Biological Age (Levine Phenotypic Age) on Chemotoxicity.

Timepoints Baseline (T0); Change over Time (T1–T0): From Baseline to 6 Months Post-Chemotherapy	Unadjusted Models ^a^		Adjusted Models ^a,b^	
	OR (95% CI)	Wald, *p*	aOR (95% CI)	Wald, *p*
**Global Chemotoxicity**				
Levine Phenotypic Age at T0	1.03 (1.02, 1.04)	73.29, **<0.001**	1.27 (1.22, 1.32)	72.30, **<0.001**
Changes in Levine Phenotypic Age	2.70 (2.42, 2.97)	110.58, **<0.001**	2.74 (2.45, 3.04)	336.96, **<0.001**
Age Acceleration (Differences from Biological Age to Chronological Age) at T0	1.30 (1.21, 1.31)	131.52, **<0.001**	1.27 (1.22, 1.32)	137.28, **<0.001**
Changes in Age Acceleration	2.70 (2.41, 2.97)	346.79, **<0.001**	2.74 (2.45, 3.05)	336.19, **<0.001**
**GI Chemotoxicity**				
Levine Phenotypic Age at T0	1.03 (1.01, 1.04)	7.47, **0.006**	1.03 (1.01, 1.05)	5.33, **0.021**
Changes in Levine Phenotypic Age	1.12 (1.04, 1.22)	8.42, **0.004**	1.10 (1.01, 1.20)	5.66, **0.042**
Age Acceleration (Differences from Biological Age to Chronological Age) at T0	1.02 (0.99, 1.04)	3.14, 0.076	1.03 (0.94, 1.05)	3.46, 0.059
Changes in Age Acceleration	1.12 (1.04, 1.22)	8.42, **0.004**	1.10 (1.02, 1.20)	5.66, **0.042**
**Hematological Chemotoxicity**				
Levine Phenotypic Age at T0	1.01 (1.01, 1.03)	6.73, **0.010**	1.06 (1.03, 1.08)	33.03, **<0.001**
Changes in Levine Phenotypic Age	1.17 (1.09, 1.26)	18.18, **<0.001**	1.15 (1.06, 1.24)	29.01, **0.005**
Age Acceleration (Differences from Biological Age to Chronological Age) at T0	1.06 (1.04, 1.08)	25.91, **<0.001**	1.06 (1.03, 1.08)	23.02, **<0.001**
Changes in Age Acceleration	1.17 (1.09, 1.26)	18.22, **<0.001**	1.15 (1.05, 1.22)	13.12, **<0.001**

Note: *p*-values are bold if they are <0.05. ^a^ When testing baseline biological age variables, we did not adjust for changes in biological age variables. When testing changes in biological age variables, we adjusted baseline biological age variables as confounders for all regression models. ^b^ For adjusted models, we controlled age groups, employment status, cancer stages, chemotherapy regimens, comorbidities, marital status, and ADI.

## Data Availability

The datasets analyzed in this study are not publicly available due to privacy restrictions but can be accessed by qualified researchers upon reasonable request to the corresponding author. As a secondary analysis of existing de-identified EHR data, our study did not generate new datasets. However, the dataset contains potentially identifiable information (including zip codes and ages above 80) that could risk patient re-identification under HIPAA regulations. Access requires approval through Ohio State University’s formal data request process, including execution of a Data Use Agreement and institutional review board approval.

## Abbreviations

The following abbreviations are used in this manuscript:

ADI	Area Deprivation Index
aOR	Adjusted Odds Ratio
BMI	Body Mass Index
CBC	Complete Blood Count
CAPEOX	Capecitabine, Oxaliplatin
CI	Confidence Interval
CRC	Colorectal Cancer
CTCAE	Common Terminology Criteria for Adverse Events
EHR	Electronic Health Record
5-FU	5-Fluorouracil
FOLFIRI	Folinic Acid, Fluorouracil, Irinotecan
FOLFOX	Folinic Acid, Fluorouracil, Oxaliplatin
GI	Gastrointestinal
HIPAA	Health Insurance Portability and Accountability Act
ICD-9/10	International Classification of Diseases, 9th/10th Revisions
IRB	Institutional Review Board
OR	Odds Ratio
ROS	Reactive Oxygen Species
SASP	Senescence-Associated Secretory Phenotype
SD	Standard Deviation
SDOH	Social Determinants of Health
SE	Standard Error

## References

[1] Siegel R.L., Kratzer T.B., Giaquinto A.N., Sung H., Jemal A. (2025). Cancer statistics, 2025. CA Cancer J. Clin..

[2] Siegel R.L., Wagle N.S., Cercek A., Smith R.A., Jemal A. (2023). Colorectal cancer statistics, 2023. CA Cancer J. Clin..

[3] Miller K.D., Nogueira L., Devasia T., Mariotto A.B., Yabroff K.R., Jemal A., Kramer J., Siegel R.L. (2022). Cancer treatment and survivorship statistics, 2022. CA Cancer J. Clin..

[4] Han C.J., Ning X., Burd C.E., Spakowicz D.J., Tounkara F., Kalady M.F., Noonan A.M., McCabe S., Von Ah D. (2024). Chemotoxicity and Associated Risk Factors in Colorectal Cancer: A Systematic Review and Meta-Analysis. Cancers.

[5] Rutherford C., Müller F., Faiz N., King M.T., White K. (2020). Patient-reported outcomes and experiences from the perspective of colorectal cancer survivors: Meta-synthesis of qualitative studies. J. Patient-Rep. Outcomes.

[6] Han C.J., Reding K.W., Kalady M.F., Yung R., Greenlee H., Paskett E.D., Cancela M.D.C. (2023). Factors associated with long-term gastrointestinal symptoms in colorectal cancer survivors in the women’s health initiatives (WHI study). PLoS ONE.

[7] Lee C.S., Ryan E.J., Doherty G.A. (2014). Gastro-intestinal toxicity of chemotherapeutics in colorectal cancer: The role of inflammation. World J. Gastroenterol..

[8] McWhirter D., Kitteringham N., Jones R.P., Malik H., Park K., Palmer D. (2013). Chemotherapy induced hepatotoxicity in metastatic colorectal cancer: A review of mechanisms and outcomes. Crit. Rev. Oncol..

[9] Abraham S., Parekh J., Lee S., Afrin H., Rozenblit M., Blenman K.R.M., Perry R.J., Ferrucci L.M., Liu J., Irwin M.L. (2025). Accelerated Aging in Cancer and Cancer Treatment: Current Status of Biomarkers. Cancer Med..

[10] Wang S., Prizment A., Thyagarajan B., Blaes A. (2021). Cancer treatment-induced accelerated aging in cancer survivors: Biology and assessment. Cancers.

[11] Sedrak M.S., Kirkland J.L., Tchkonia T., Kuchel G.A. (2021). Accelerated aging in older cancer survivors. J. Am. Geriatr. Soc..

[12] Berben L., Floris G., Wildiers H., Hatse S. (2021). Cancer and Aging: Two Tightly Interconnected Biological Processes. Cancers.

[13] Westrick A.C., Langa K.M., Eastman M., Ospina-Romero M., Mullins M.A., Kobayashi L.C. (2022). Functional aging trajectories of older cancer survivors: A latent growth analysis of the US Health and Retirement Study. J. Cancer Surviv..

[14] Carroll J.E., Bower J.E., Ganz P.A. (2021). Cancer-related accelerated ageing and biobehavioural modifiers: A framework for research and clinical care. Nat. Rev. Clin. Oncol..

[15] Guida J.L., Agurs-Collins T., A Ahles T., Campisi J., Dale W., Demark-Wahnefried W., Dietrich J., Fuldner R., Gallicchio L., A Green P. (2021). Strategies to Prevent or Remediate Cancer and Treatment-Related Aging. JNCI J. Natl. Cancer Inst..

[16] Guo J., Huang X., Dou L., Yan M., Shen T., Tang W., Li J. (2022). Aging and aging-related diseases: From molecular mechanisms to interventions and treatments. Signal Transduct. Target. Ther..

[17] Prasanna P.G., Citrin D.E., Hildesheim J., Ahmed M.M., Venkatachalam S., Riscuta G., Xi D., Zheng G., van Deursen J., Goronzy J. (2021). Therapy-Induced Senescence: Opportunities to Improve Anticancer Therapy. JNCI J. Natl. Cancer Inst..

[18] Jurkovicova D., Neophytou C.M., Gašparović A.Č., Gonçalves A.C. (2022). DNA Damage Response in Cancer Therapy and Resistance: Challenges and Opportunities. Int. J. Mol. Sci..

[19] Nikita N., Sun Z., Sharma S., Shaver A., Seewaldt V., Lu-Yao G. (2025). Epigenetic Landscapes of Aging in Breast Cancer Survivors: Unraveling the Impact of Therapeutic Interventions—A Scoping Review. Cancers.

[20] Bhatia R., Holtan S., Jurdi N.E., Prizment A., Blaes A. (2022). Do cancer and cancer treatments accelerate aging?. Curr. Oncol. Rep..

[21] Wennberg A.M., Matthews A., Talbäck M., Ebeling M., Ek S., Feychting M., Modig K. (2023). Frailty Among Breast Cancer Survivors: Evidence From Swedish Population Data. Am. J. Epidemiol..

[22] Zahnreich S., Schmidberger H. (2021). Childhood Cancer: Occurrence, Treatment and Risk of Second Primary Malignancies. Cancers.

[23] Lucas F., Pennell M., Huang Y., Benson D.M., Efebera Y.A., Chaudhry M., Hughes T., Woyach J.A., Byrd J.C., Zhang S. (2020). T Cell Transcriptional Profiling and Immunophenotyping Uncover LAG3 as a Potential Significant Target of Immune Modulation in Multiple Myeloma. Biol. Blood Marrow Transplant..

[24] Wood W.A., Krishnamurthy J., Mitin N., Torrice C., Parker J.S., Snavely A.C., Shea T.C., Serody J.S., Sharpless N.E. (2016). Chemotherapy and Stem Cell Transplantation Increase p16 INK4a Expression, a Biomarker of T-cell Aging. EBioMedicine.

[25] E Burd C., Peng J., Laskowski B.F., Hollyfield J.L., Zhang S., Fadda P., Yu L., Andridge R.R., Kiecolt-Glaser J.K., Le Couteur D. (2020). Association of Epigenetic Age and *p16 INK4a* With Markers of T-Cell Composition in a Healthy Cohort. J. Gerontol. Ser. A.

[26] Ji J., Sun C.-L., LeVee A.A., Sedrak M.S. (2024). Accelerated epigenetic aging and risk of chemotoxicity in older adults with early breast cancer. J. Clin. Oncol..

[27] Xiao C., Miller A.H., Peng G., Levine M.E., Conneely K.N., Zhao H., Eldridge R.C., Wommack E.C., Jeon S., Higgins K.A. (2021). Association of Epigenetic Age Acceleration With Risk Factors, Survival, and Quality of Life in Patients With Head and Neck Cancer. Int. J. Radiat. Oncol..

[28] Liu X., Wang Y., Huang Y., Lin C., Xu B., Zeng Y., Chen P., Liu X., Huang Y. (2025). Association of phenotypic age acceleration with all-cause and cause-specific mortality among U.S. cancer survivors: A retrospective cohort study. BMC Cancer.

[29] Chen R., Wang Y., Zhang S., Bulloch G., Zhang J., Liao H., Shang X., Clark M., Peng Q., Ge Z. (2023). Biomarkers of ageing: Current state-of-art, challenges, and opportunities. MedComm—Future Med..

[30] Levine M.E., Lu A.T., Quach A., Chen B.H., Assimes T.L., Bandinelli S., Hou L., Baccarelli A.A., Stewart J.D., Li Y. (2018). An epigenetic biomarker of aging for lifespan and healthspan. Aging.

[31] Rosenzweig M.Q., Althouse A.D., Sabik L., Arnold R., Chu E., Smith T.J., Smith K., White D., Schenker Y. (2021). The Association Between Area Deprivation Index and Patient-Reported Outcomes in Patients with Advanced Cancer. Health Equity.

[32] Cheng E., Soulos P.R., Irwin M.L., Feliciano E.M.C., Presley C.J., Fuchs C.S., Meyerhardt J.A., Gross C.P. (2021). Neighborhood and Individual Socioeconomic Disadvantage and Survival Among Patients With Nonmetastatic Common Cancers. JAMA Netw. Open.

[33] National Cancer Institute, National Institutes of Health, US Department of Health and Human Services Common Terminology Criteria for Adverse Events (CTCAE) Version 5.0. https://dctd.cancer.gov/research/ctep-trials/for-sites/adverse-events/ctcae-v5-5x7.pdf.

[34] Chen S.-Y., Feng Z., Yi X. (2017). A general introduction to adjustment for multiple comparisons. J. Thorac. Dis..

[35] Wang B., Kohli J., Demaria M. (2020). Senescent Cells in Cancer Therapy: Friends or Foes?. Trends Cancer.

[36] Brouwers B., Hatse S., Lago L.D., Neven P., Vuylsteke P., Dalmasso B., Debrock G., Bulck H.V.D., Smeets A., Bechter O. (2016). The impact of adjuvant chemotherapy in older breast cancer patients on clinical and biological aging parameters. Oncotarget.

[37] Sebastiani P., Thyagarajan B., Sun F., Schupf N., Newman A.B., Montano M., Perls T.T. (2017). Biomarker signatures of aging. Aging Cell.

[38] Shachar S.S., Deal A.M., E Reeder-Hayes K., A Nyrop K., Mitin N., Anders C.K., A Carey L., Dees E.C., A Jolly T., Kimmick G.G. (2020). Effects of Breast Cancer Adjuvant Chemotherapy Regimens on Expression of the Aging Biomarker, *p16INK4a*. JNCI Cancer Spectr..

[39] Liu C.H., Abrams N.D., Carrick D.M., Chander P., Dwyer J., Hamlet M.R.J., Macchiarini F., PrabhuDas M., Shen G.L., Tandon P. (2017). Biomarkers of chronic inflammation in disease development and prevention: Challenges and opportunities. Nat. Immunol..

[40] Jakubczyk K., Dec K., Kałduńska J., Kawczuga D., Kochman J., Janda K. (2020). Reactive oxygen species—Sources, functions, oxidative damage. Pol. Merkur. Lekarski..

[41] Wang B., Han J., Elisseeff J.H., Demaria M. (2024). The senescence-associated secretory phenotype and its physiological and pathological implications. Nat. Rev. Mol. Cell Biol..

[42] Sanoff H.K., Deal A.M., Krishnamurthy J., Torrice C., Dillon P., Sorrentino J., Ibrahim J.G., Jolly T.A., Williams G., Carey L.A. (2014). Effect of Cytotoxic Chemotherapy on Markers of Molecular Age in Patients With Breast Cancer. JNCI J. Natl. Cancer Inst..

[43] Barchitta M., Maugeri A., Li Destri G., Basile G., Agodi A. (2019). Epigenetic Biomarkers in Colorectal Cancer Patients Receiving Adjuvant or Neoadjuvant Therapy: A Systematic Review of Epidemiological Studies. Int. J. Mol. Sci..

[44] Read B., Sylla P. (2020). Aggressive Colorectal Cancer in the Young. Clin. Colon Rectal Surg..

[45] Thomas A., Belsky D.W., Gu Y., Duque G. (2023). Healthy Lifestyle Behaviors and Biological Aging in the U.S. National Health and Nutrition Examination Surveys 1999–2018. J. Gerontol. Ser. A.

[46] Oblak L., van der Zaag J., Higgins-Chen A.T., Levine M.E., Boks M.P. (2021). A systematic review of biological, social and environmental factors associated with epigenetic clock acceleration. Ageing Res. Rev..

[47] Quintela-Fandino M., Soberon N., Lluch A., Manso L., Calvo I., Cortes J., Moreno-Antón F., Gil-Gil M., Martinez-Jánez N., Gonzalez-Martin A. (2017). Critically short telomeres and toxicity of chemotherapy in early breast cancer. Oncotarget.

[48] M’kAcher R., Bennaceur-Griscelli A., Girinsky T., Koscielny S., Delhommeau F., Dossou J., Violot D., Leclercq E., Courtier M.H., Béron-Gaillard N. (2007). Telomere Shortening and Associated Chromosomal Instability in Peripheral Blood Lymphocytes of Patients With Hodgkin’s Lymphoma Prior to Any Treatment Are Predictive of Second Cancers. Int. J. Radiat. Oncol..

[49] Falandry C., Horard B., Bruyas A., Legouffe E., Cretin J., Meunier J., Alexandre J., Delecroix V., Fabbro M., Certain M.-N. (2015). Telomere length is a prognostic biomarker in elderly advanced ovarian cancer patients: A multicenter GINECO study. Aging.

[50] Mitin N., Nyrop K.A., Strum S.L., Knecht A., Carey L.A., Reeder-Hayes K.E., Dees E.C., Jolly T.A., Kimmick G.G., Karuturi M.S. (2022). A biomarker of aging, p16, predicts peripheral neuropathy in women receiving adjuvant taxanes for breast cancer. npj Breast Cancer.

[51] DeMaria M., O’Leary M.N., Chang J., Shao L., Liu S., Alimirah F., Koenig K., Le C., Mitin N., Deal A.M. (2017). Cellular Senescence Promotes Adverse Effects of Chemotherapy and Cancer Relapse. Cancer Discov..

[52] Rosko A.E., Elsaid M.I., Woyach J., Islam N., Lepola N., Urrutia J., Christian L.M., Presley C., Mims A., Burd C.E. (2024). Determining the relationship of p16INK4a and additional molecular markers of aging with clinical frailty in hematologic malignancy. J. Cancer Surviv..

[53] Ros M., Carrascosa J.M. (2020). Current nutritional and pharmacological anti-aging interventions. Biochim. Biophys. Acta (BBA)—Mol. Basis Dis..

